# Charge Reversal of the Uppermost Arginine in Sliding Helix S4-I Affects Gating of Cardiac Sodium Channel

**DOI:** 10.3390/ijms26020712

**Published:** 2025-01-16

**Authors:** Olga E. Kulichik, Anastasia K. Zaytseva, Anna A. Kostareva, Boris S. Zhorov

**Affiliations:** 1 Almazov National Medical Research Centre, 197341 St. Petersburg, Russia; olya.kulichik@mail.ru (O.E.K.); zaytseva.anastasia.zak@gmail.com (A.K.Z.); anna.kostareva@ki.se (A.A.K.); 2 Sechenov Institute of Evolutionary Physiology & Biochemistry, Russian Academy of Sciences, 194223 St. Petersburg, Russia; 3 Department of Women’s and Children’s Health and Center for Molecular Medicine, Karolinska Institutet, 171 76 Stockholm, Sweden; 4 Department of Biochemistry and Biomedical Sciences, McMaster University, Hamilton, ON L8S 4K1, Canada

**Keywords:** arrhythmia, intersegment contacts, molecular modeling, Nav1.5, sodium channelopathies, patch-clamp

## Abstract

Several mutations of the uppermost arginine, R219, in the voltage-sensing sliding helix S4_I_ of cardiac sodium channel Nav1.5 are reported in the ClinVar databases, but the clinical significance of the respective variants is unknown (VUSs). AlphaFold 3 models predicted a significant downshift of S4_I_ in the R219C VUS. Analogous downshift S4_I_, upon its in silico deactivation, resulted in a salt bridge between R219 and the uppermost glutamate, E161, in helix S2_I_. To understand how salt bridge elimination affects biophysical characteristics, we generated mutant channel R219E, expressed it in the HEK293-T cells, and employed the patch-clamp method in a whole-cell configuration. Mutation R219E did not change the peak current density but shortened time to the peak current at several potentials, significantly enhanced activation, enhanced steady-state inactivation and steady-state fast inactivation, and slowed recovery from inactivation. Taken together, these data suggest that mutation R219E destabilized the resting state of Nav1.5. Cardiac syndromes associated with mutations R219P/H/C/P or E161Q/K are consistent with the observed changes of biophysical characteristics of mutant channel R219E suggesting pathogenicity of the respective VUSs, as well as ClinVar-reported VUSs involving arginine or glutamate in homologous positions of several Nav1.5 paralogs.

## 1. Introduction

Cardiac voltage-gated sodium channels (Nav1.5) are responsible for the initiation and propagation of action potentials (AP) in cardiomyocytes by allowing the rapid entry of sodium ions into the cell during phase 0 [1]. The Nav1.5 channel has a pore-forming α subunit of 220 kDa [2] and one or more regulatory β subunits of 30–40 kDa [3]. The α subunit, which is encoded by the *SCN5A* gene localized in chromosome 3p21 [4], is a pseudo hetero-tetramer of four homologous repeats (I–IV). Each repeat contains six transmembrane α-helical segments, S1–S6. Helices S1 to S4 form a voltage-sensing domain (VSD). Helices S5, S6, and a large extracellular membrane re-entering P-loop between S5 and S6 contribute a quarter to the pore domain (PD) [5,6,7]. Each P-loop contains ascending (P1) and descending (P2) helices with a selectivity filter residue between them [8]. A voltage-sensing siding helix, S4, features positively charged Arg or Lys residues, which collectively move the helix across the membrane in response to changes of the membrane voltage. Navs exist in several states. At the resting membrane potential (−90 to −80 mV), the channels are in the resting state [9]. Membrane depolarization activates VSD_I_, VSD_II_, and VSD_III_, and the activation gate in the pore domain opens up. Subsequent activation of VSD_IV_ leads to fast inactivation [10]. With prolonged or repeated depolarization, the channels enter slow inactivated state(s). Upon recovery from inactivation, the channel transits to deactivated and then to the resting state; see [11] for a review.

According to the World Health Organization, by 2030, the number of diagnosed cases of cardiovascular diseases may increase by 40% relative to 2022 [12]. Thus, the pathology of the cardiovascular system, in particular arrhythmias, is an urgent problem in modern healthcare. Pathogenic variants of the *SCN5A* gene are associated with the development of arrhythmogenic syndromes. Missense variants are predominantly present in the heterozygous state. *SCN5A*-associated syndromes include Brugada syndrome type 1 (BrS1), long QT syndrome type 3 (LQT3), sick sinus syndrome, progressive cardiac conduction disorder (Lev–Lenegre syndrome), and other diseases [13]. ClinVar [14] and other databases accumulate data on inherited variants of the *SCN5A* gene. Most of the identified missense variants are observed in single patients and, according to the American College of Medical Genetics classification, they are variants of unknown significance, VUSs [15]. Information about the clinical significance of a specific genetic variant is essential for proper diagnosis, risk stratification, and patient management. Functional studies allow for the determination of biophysical mechanisms of arrhythmias. The patch-clamp technique allows the biophysical properties of Nav channels to be studied and their functional activity to be evaluated.

Pathogenic genetic variants can stabilize or destabilize various channel states. Respective changes in the equilibrium between different Nav1.5 states may affect channel conductance, duration and amplitude of the peak and late sodium currents, or disrupt inactivation, resulting in prolonged sodium currents during the AP plateau phase [1]. These changes can affect the normal heart physiology and lead to formation of arrhythmogenic foci. Moreover, disturbances in the structure of Nav1.5 can lead to both gain-of-function (GoF) and loss-of-function (LoF) phenotypes [16]. The GoF syndromes are associated with delayed inactivation, accelerated activation, or accelerated recovery from the inactivation. As a result, later repolarization increases the duration of the ventricular AP and could increase the QT interval [9]. A decreased channel activity can cause stabilization of the inactivated or resting state and destabilization of the activated state. As a result, myocardial excitability decreases and conduction slows down [9].

The vast majority of mutations, which are localized in VSDs, are described in the ClinVar database as VUSs. Functional studies of mutant channels are necessary to understand molecular mechanisms of possible channel dysfunction associated with such mutations. If a functional study demonstrates significant changes in the biophysical characteristics of a mutant channel, VUSs in the respective position may be reclassified as likely pathogenic (LP) variants.

In this study, we focus on the uppermost arginine in the voltage-sensing helix S4 of VSD_I_ (R1_S4_I_ or R1). Various mutations of this arginine in the Nav1.5 channel and its paralogues are reported in the ClinVar database as VUSs. We first analyzed state-dependent contacts of R1 in helix S4_I_ in experimental structures of Navs, in AlphaFold 3 (AF3) models of VSD_I_ variants, and in in silico deactivated S4_I_ in the WT and R219E mutant channels. These structural data strongly suggest a state-dependent salt bridge, R219:E161. We further generated mutant channel R219E, expressed it in the HEK293-T cells, and demonstrated significant changes in various biophysical characteristics of the mutant channel versus the WT channel. Based on these data, we proposed an atomic mechanism of the observed dysfunction of the Nav1.5-R219E mutant channel. In view of significant changes in the biophysical characteristics of the mutant channel R219E, we propose that fifteen VUSs in respective positions of Nav1.5 and its sodium channel paralogs may be reclassified as P/LP variants.

## 2. Results

### 2.1. Arginine R1 in Helix S4_I_ (R1_S4_I_) and Glutamate E1 in Helix S2_I_ (E1_S2_I_) of VSD_I_ in Experimental and Computed Structures of Nav1.x Channels

**Figure 1:**
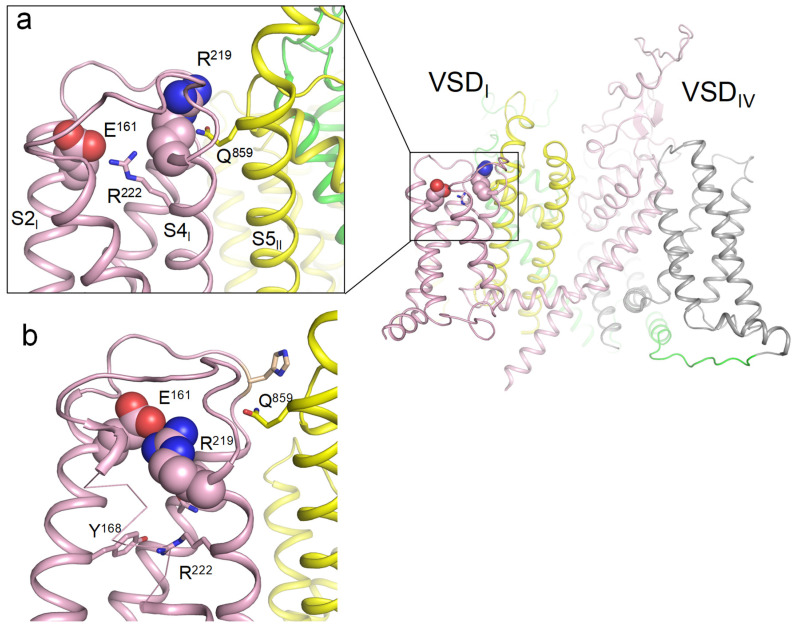
(**a**) Membrane view of the cryoEM structure with activated VSDs (PDB ID: 6lqa). Repeats I, II, III, and IV are shown in pink, yellow, green, and gray, respectively. R219 forms a polar contact with glutamine Q859 in helix S5_I_ of the pore domain, while glutamate E161 forms a salt bridge with arginine R222. (**b**) Deactivated VSD_I_ visualized using coordinates from [18]. R219 forms a salt bridge with glutamate E161. Mutation R219E would cause electrostatic repulsion between the two glutamates.

In the cryoEM structure of channel hNav1.5 (PDB ID: 6lqa), R1_S4_I_ (R219) forms a polar contact with Q859 in helix S5_II_ of the pore domain, whereas E1_S2_I_ (E161) forms a salt bridge with R2_S4_I_ (R222) (Figure 1a). Among other cryoEM structures of Nav1.x channels with activated VSDs, R1_S4_I_ interacts with E1_S2_I_ only in the Nav1.8 channel (PDB ID: 7wel, 7we4, 7wfr). Thus, available cryoEM structures of eukaryotic sodium channels with activated VSDs do not provide a consensus view on contacts of ionizable residues in the extracellular half of VSD_I_.

Earlier, we used crystal structures of the prokaryotic sodium channel NavAb with the sliding helices S4 in the activated (PDB ID: 6p6x) and resting (PDB ID: 6p6w) states [17] to build homology models of the hNav1.5 channel with resting and activated VSDs [18]. In our resting-state model of hNav1.5, R1_S4_I_ is salt-bridged to E1_S2_I_ (Figure 1b). Respective arginine and glutamate residues are present in VSD_I_ of all Nav1.x channels, indicating their functional importance.

**Figure 2:**
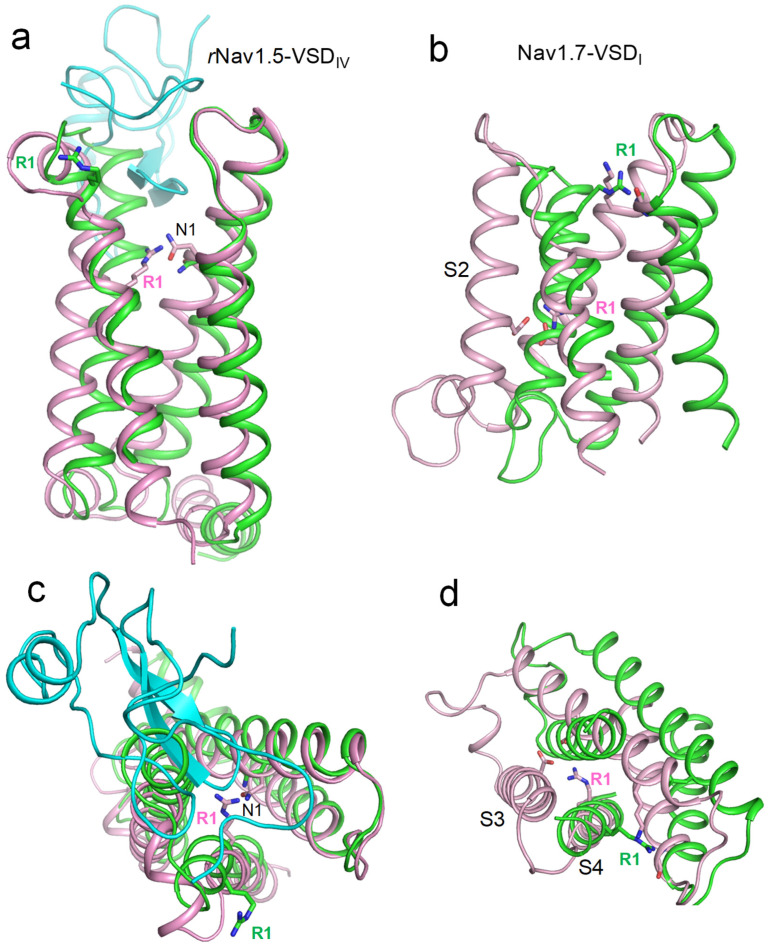
Activated and deactivated VSDs in cryoEM structures of toxin-bound rNav1.5 and mutated hNav1.7. Shown are membrane (**a**,**b**) and extracellular (**c**,**d**) views of 3D-aligned cryo-EM structures with activated (green) and deactivated (magenta) VSDs. Upon VSD deactivation, state-dependent contacts of the uppermost arginines (R1) contribute to VSD stabilization. (**a**,**c**) rNav1.5 in the apo state (PDB ID: 6uz3) and in complex with gating-modifying toxin LQHIII (PDB ID: 7xsu). The toxin (cyan) downshifts S4_IV_ by two helical turns (**a**). It also twists S4_IV_ anticlockwise (**c**) and somewhat shifts helices in the membrane plane (**c**). In the toxin-bound deactivated state, R1 interacts with the uppermost D1_S2, which sequentially aligns with Nav1.5 glutamate E1_S2_I_. (**c**,**d**) WT channel hNav1.7 (PDB ID: 7xvf) and mutant channel with deactivated VSD_I_ (PDB ID: 7xve). In the deactivated VSD_I_, R1 forms salt bridges with an asparagine in helix S3 and a glutamate in helix S2.

An important piece of evidence regarding state-dependent contacts of voltage-sensing arginines R1 in Nav1.5 channels was provided by William Catterall and colleagues [19]. The authors obtained cryoEM structures of the rat channel Nav1.5 in the apo-state and in complex with the deathstalker scorpion α-toxin LqhIII that traps VSD_IV_ in the resting state. A comparison of the 3D aligned structures of rNav1.5 channel in the apo and toxin-bound states showed that the toxin binding downshifted the backbone atoms of R1_S4_IV_ by two helical turns as compared with the apo channel (Figure 2a,c). In the toxin-bound structure, the side chain of R1_S4_IV_ was unresolved, implying its high flexibility. In Monte Carlo (MC) minimized models of this structure, R1_S4_IV_ adopted two energetically optimal orientations, forming salt bridges either with E1_S3_IV_ or E2_S2_IV_.

The CryoEM structure of the hNav1.7 channel with 11 engineered point mutations (PDB ID: 7xve) [20] shows VSD_I_ in a completely deactivated, down state (Figure 2b,d). In this structure, the side chain of R1_S4_I_ points in the cytoplasmic direction, forming state-dependent salt bridges with glutamate residues in the cytoplasmic halves of helices S2_I_ and S3_I_. However, this structure does not rule out the possibility of a salt bridge between R1_S4_I_ and E1_S2_I_ in the hNav1.5 channel. Indeed, in the mutated channel hNav1.7, E1_S2_I_ is substituted with lysine that repels rather than attracts R1_S4_I_. Furthermore, the hNav1.5 channel lacks an acidic residue in the cytoplasmic half of S3_I_, which, together with E2_S2_I_, attracts R1_S4_I_ in the cryoEM structure of mutant channel hNav1.7.

### 2.2. AlphaFold 3 Structures of VSD_I_ in WT Nav1.5 and ClinVar-Reported Variants of R1_S4_I_ and E1_S2_I_

**Figure 3:**
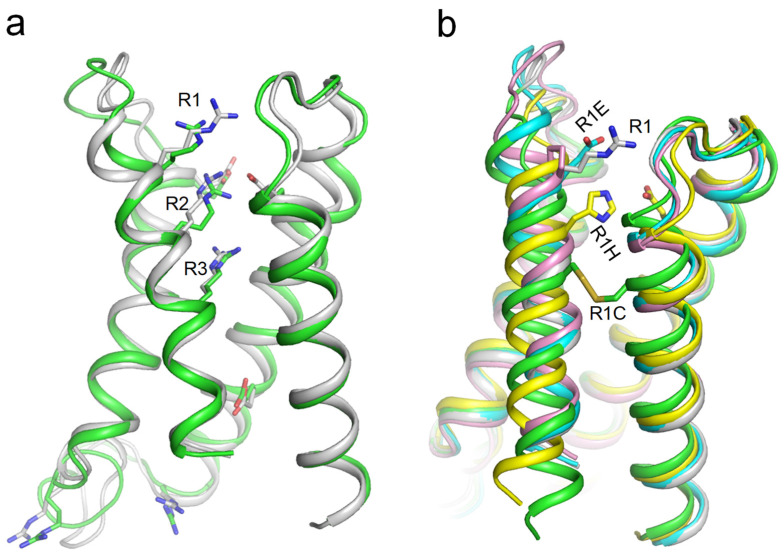
(**a**) VSD_I_ in cryo-EM structure of hNav1.5 (PDB ID: 6lqa, green) and AF3 model of lone VSD_I_ from hNav1.5 (gray). Arginine and glutamate residues are shown as sticks. (**b**) 3D aligned VSD_I_ models generated by AF3: WT (gray), R1E (cyan), R1H (yellow), R1C (green), and R1P (magenta). R1 in the WT model and Glu in the R1E model do not make intersegment contacts. Cys in the R1C model formed a disulfide bond with a cysteine in helix S1, causing a downshift of S4_I_ by two helical turns.

We generated AF3 models of VSD_I_ in the WT channel hNav1.5 and in four mutant channels where R1_S4_I_ is substituted with Glu, Cys, His, or Pro and 3D-aligned the models with PyMol. Glutamate or proline substitutions of R1_S4_I_ resulted in only minimal changes in the VSD_I_ folding (Figure 3b). In contrast, the cysteine substitution of R1_S4_I_ down-shifted the cysteine by two helical turns, and caused a disulfide bond with a cysteine in the middle of helix S1_I_. Helix S4_I_ adopted a deactivated position (Figure 3b), which was seen in the cryoEM structure hNav1.7 with eleven engineered residues in VSD_I_ (Figure 2b). A significant downshift was also predicted for the histidine substitution of R1_S4_I_, likely due to attraction of the protonated histidine to the uppermost glutamate E1_S2_I_ (Figure 3b). Thus, AF3 models do predict significant downshifts of S4_I_ in mutant channels where R1_S4_I_ is substituted by cysteine or histidine, suggesting dysfunction of these ClinVar-reported variants of hNav1.5.

### 2.3. In Silico Deactivating Lone VSD_I_ of the WT Channel Nav1.5 and Mutant Channel R219E

**Figure 4:**
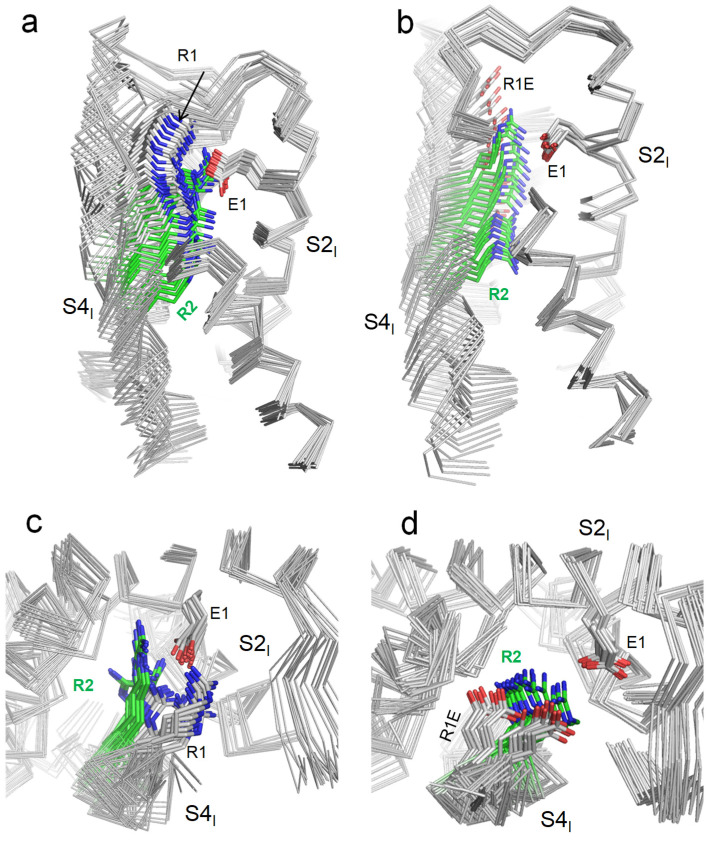
In silico deactivation of VSD_I_ in the WT channel hNav1.5 (**a**,**c**) and mutant channel R219E (**b**,**d**). The backbones are shown by ribbons, with some parts removed for clarity. (**a**,**b**) membrane views. (**c**,**d**) Extracellular views. Note green color of sidechain carbon atoms in R2.

To model deactivation of VSD_I_ in the WT and R219E channels, we in silico downshifted S4_I_ as described in the methods section. Downshifting of S4_I_ in the WT channel yielded 21 MC-minimized structures. The strongest attraction between R219 and E161 was found at the final step of the trajectory, where these residues were salt-bridged (Figure 4a). At the extracellular view of the 21 structures, S4_I_ turned anticlockwise (Figure 4b), resembling the anticlockwise turns in the cryoEM structures of toxin-deactivated S4_IV_ of rNav1.5 (Figure 2c) and deactivated VSD_I_ in the mutant channel hNav1.7 (Figure 2d).

Upon in silico deactivation of VSD_I_ in the mutant channel R219E (Figure 4b,d), carbon atoms of the COO groups in R219E and E161 were 9.7 Å apart in the first step of the S4_I_ downshifting trajectory. The distance decreased to 5.1 Å in step 14 and increased to 8.1 Å in the final step, 21. In step 14, the strong repulsion between the two carboxylates was compensated by their electrostatic attraction to R222. As in the WT VSD_I_, S4_I_ turned anticlockwise (when viewed from the extracellular space).

The above analysis of cryoEM structures and our molecular models suggests that, in the resting state of hNav1.5_VSD_I_, R219 was salt-bridged with glutamate E161. Mutations of R219 in ClinVar-reported variants eliminated this salt bridge and may have affected the channel gating. The strongest impact on the gating was expected for mutant channel R219E where the electrostatic repulsion between the engineered and native glutamates would destabilize the resting state of VSD_I_. With this idea in mind, we generated mutant channel R219E and explored its biophysical properties.

### 2.4. Biophysical Characteristics of the WT and R219E Channels

**Figure 5:**
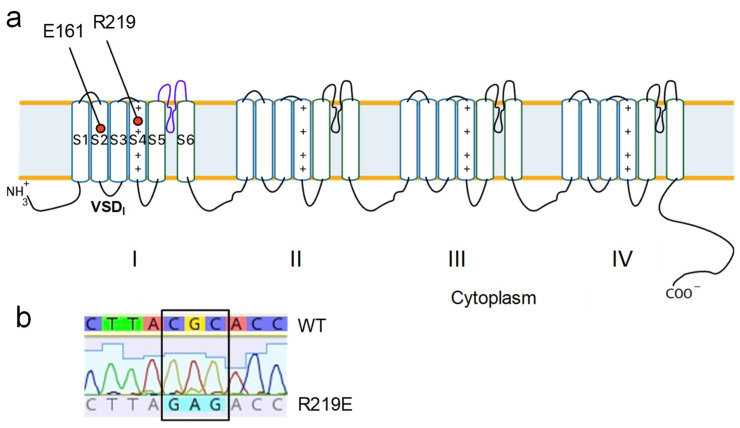
Generating mutant construct R219E-Nav1.5. (**a**) E161 and R219 in the topological model of the Nav1.5 channel. (**b**) Results of site-specific mutagenesis verified using the Sanger sequencing method.

We generated mutant construction R219E-pcDNA3.1 using site-directed mutagenesis via PCR amplification (Figure 5a). The substitution was verified with Sanger sequencing (Figure 5b). The plasmid vector with the substitution was expressed in the HEK293-T cell culture using GFP as a marker of transfection efficiency.

**Figure 6:**
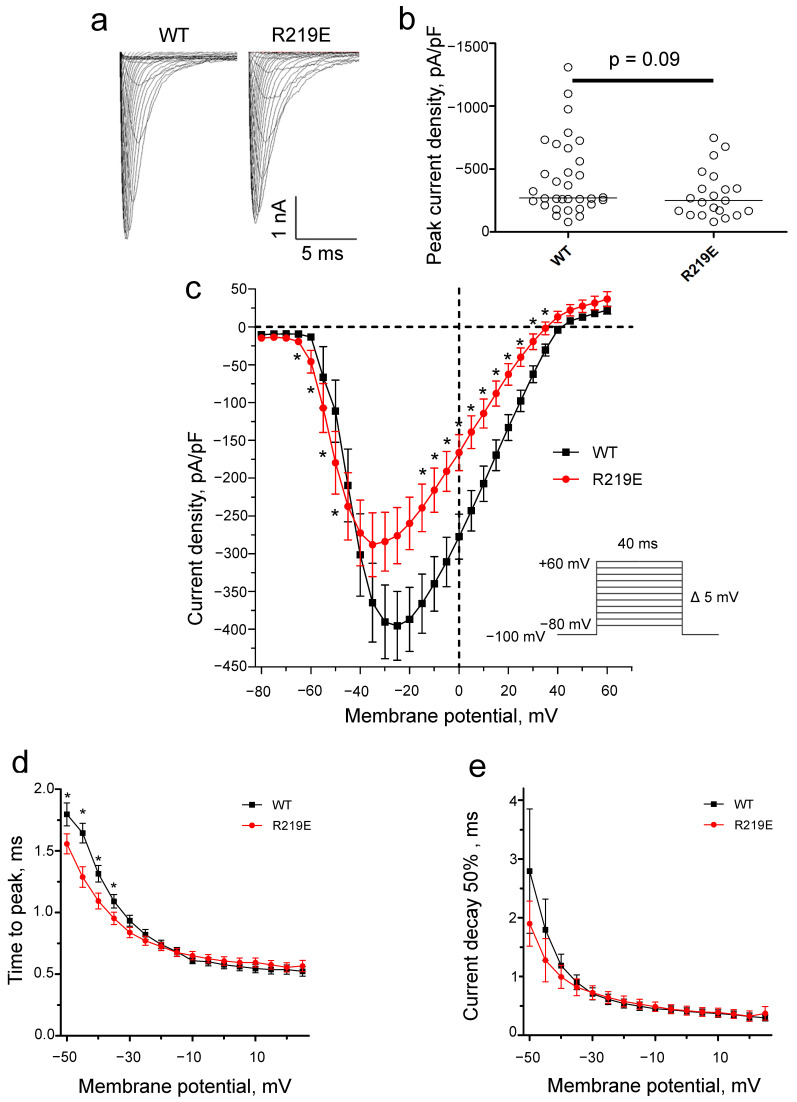
Changes in Na^+^ current due to mutation of R219E. (**a**) Representative recordings of Na^+^ current for the WT and mutant channel R219E. (**b**) Distribution of Na^+^ current density at peak values. There was no significant difference for the WT and R219E channels. Each circle corresponds to an experiment with one cell (WT: n = 32; R219E: n = 21). (**c**) Volt-ampere characteristics of Na^+^ currents. Mutation R219E caused significant changes in the current density at some voltages. Asterisks (*) indicate significantly different values. (**d**) The voltage dependence of time to peak Na^+^ current for the WT and R219E channels. Mutation R219E caused a marked reduction in time to peak in the range from −50 to −35 mV. (**e**) Time of 50% decay for WT (black squares) and R219E (red circles) channels. Mutation R219E did not significantly influence the time of 50% decay.

Mutation R219E led to functionally active sodium channels with typical currents (Figure 6a). The capacitance of cells expressing Nav1.5-R219E did not significantly differ from that of Nav1.5-WT (WT: 8.6 ± 0.5 pF, n = 32; R219E: 9.2 ± 0.8 pF, n = 21), indicating that the cell size was similar in the control and experimental groups. R219E did not significantly change the peak sodium current density (Figure 6b; Table 1), but the current density significantly decreased at potentials from −40 and +40 mV (Figure 6c). The time to peak of the R219E channel was significantly lower at potentials from −50 to −40 mV (Figure 6d), indicating the enhanced activation. Mutation R219E did not affect the current decay to 50% at potentials between −50 and −45 mV (Figure 6e).

**Figure 7:**
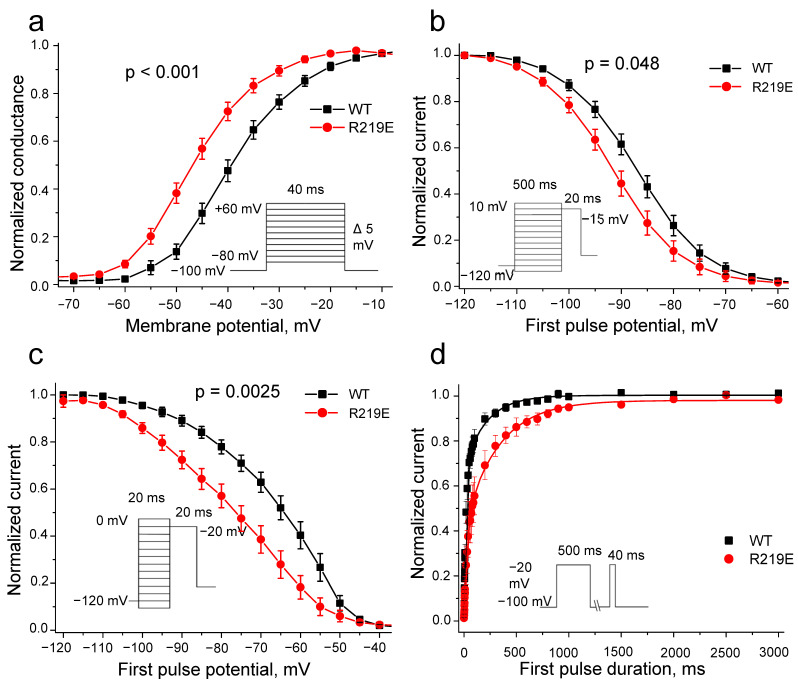
Biophysical characteristics of wild-type WT (black squares) and R219E (red circles) channels. (**a**) Steady-state activation. Mutation R219E caused a significant shift (−9.6 mV) of the steady-state activation (WT: n = 32; R219E: n = 21). (**b**) Steady-state inactivation for the WT (black squares) and R219E (red circles) channels. Mutation caused a negative shift of steady-state inactivation (WT: n = 24; R219E: n = 15). (**c**) Steady-state fast inactivation for the WT (black squares) and R219E (red circles) channels. Mutation R219E cased a significant shift of (15 mV) of the fast inactivation curve towards hyperpolarized potentials (WT: n = 14; R219E: n = 14). (**d**) Time course of recovery from inactivation. Mutation R219E markedly decelerated recovery from inactivation.

Mutation R219E caused a significant hyperpolarization shift (9.6 mV) of the steady-state activation curve (Figure 7a; Table 1), indicating an accelerated activation that is consistent with observed reduction in time to peak. Mutation R219E also statistically significantly shifted the curve of the steady-state inactivation by −5.6 mV (Figure 7b, Table 1), indicating accelerated inactivation.

Next, we explored the fast inactivation of mutant channels to determine the role of this process in the changes in steady-state inactivation and observed a significant negative shift of −15.1 mV (Figure 7c; Table 1). The changed kinetics of the steady-state inactivation may be associated with the enhanced activation (“easy on—easy off”). Thus, the observed alterations in inactivation could be explained as the consequences of the enhanced activation. The R219E channels also demonstrated slower recovery from inactivation (Figure 7d, Table 1), supporting the resting-state destabilization.

## 3. Discussion

Earlier, we proposed that R219 in the hNav1.5 channel is salt-bridged with E219 in the resting but not activated state of VSD_I_ [18]. Here, we analyzed cryoEM structures of Nav1.x channels, including two channels in which VSDs are captured in both the resting and activated states. We further deactivated VSD_I_ in silico in the WT hNav1.5 channel and R219E channel. Our analyses of cryoEM structures and computational results strongly suggest that R219 forms a salt bridge with E219 in the resting but not activated VSD_I_. To experimentally test this hypothesis, we generated mutant channel hNav1.5-R219E where the electrostatic repulsion between R219E and E161 would be strong, and explored the biophysical properties of the WT and mutant channels. We found that mutation R219E enhanced activation, inactivation, and fast inactivation. It also slowed recovery from inactivation, but did not change the peak current density.

Several Nav1.5 genetic variants involving E161 or R219 have previously been functionally characterized. Thus, mutation E161K caused a positive shift of the steady-state activation, but did not change the kinetics of recovery from the inactivation, which can decrease the cardiac excitability [13]. Other functional studies of the E161K mutant channel using the tsA201 expression model demonstrated similar changes in electrophysiological characteristics [21]. The above studies show that mutation E161K, which eliminates the salt bridge between R1_S4_I_ and E1_S2_I_, impairs activation. In contrast, in our experiments, mutation R219E accelerated the channel activation. A likely cause for this discrepancy is that E161 may form state-dependent salt bridges with two voltage sensing arginines, R219 and R222 (Figure 4a). In the mutant channel E161K, repulsion between the engineered lysine K161 and arginine R222 would obstruct the voltage-dependent shift of S4_I_ in the extracellular direction, thus retarding activation of VSD_I_. In contrast, the major effect of mutation of R219E is destabilization of the resting-state salt bridge R219:E161 in the WT channel, which facilitates channel activation. Destabilization of the resting state could also explain the observed impairment of recovery from inactivation. The curve of recovery from inactivation reflects the transition between inactivated and resting states. The channel inactivation involves activation of VSD_IV_ [11], whereas channel transition to the resting state involves deactivation of all VSDs. Therefore, the slower recovery from inactivation in the R219E mutant channel could be due to destabilization of the VSD_I_ resting (deactivated) state because of repulsion between R219E and E161.

We observed the enhanced steady state and fast inactivation of the mutant channel R219E (Figure 7b,c). The facilitated activation may accelerate inactivation due to a reduced duration of the channel activated state (“easy on—easy off”). This suggestion is supported by the shorter time to the peak current without changes in the inactivation decay characteristics (Figure 6d,e). Mutation R222Q, associated with dilated cardiomyopathy, caused enhanced activation and inactivation [22], suggesting that dysfunctions of mutant channels R219E and R222Q have similar mechanisms. Our data are consistent with a recent study in which charge-neutralizing mutations (R1Q, R2Q, and R3Q) were generated for each VSD in several Navs, and movement VSD_I_ was proposed to be rate limiting for the Nav1.5 pore opening [23]. Earlier, a similar conclusion was made for the Nav1.4 channel [10].

Some mutations beyond VSD_I_ also cause both gain-of-function (GoF) and lost-of-function (LoF) effects. Mutation T1620K in the S3-S4_IV_ extracellular loop accelerates steady-state activation, increases the slope of the steady-state inactivation, and causes a late current, i.e., both LoF and GoF [24]. Mutation R340Q in the P-loop S5-S6_I_ caused a negative shift in the steady-state activation and inactivation, as well as facilitating fast inactivation [25]. Mutation V411M accelerated activation and inactivation and caused late currents, but no changes in current density were recorded [26]. Mutations V1777M and S1904L in the C-terminal region caused a negative shift in the steady-state activation and inactivation and increased the sodium current [27,28]. Most of the above substitutions lead to complex changes in biophysical characteristics and are associated with the development of various syndromes and mixed phenotypes: BrS1, LQT3, dilated cardiomyopathy, sick sinus syndrome, etc.

Some Nav 1.5 variants involving R219 or E161 were explored in earlier biophysical studies. Thus, mutation E161K impaired activation [13,21]. Mutation R219H is proposed to cause a proton leakage current [29,30]. Five of six variants of R219 or E161, which are reported in the ClinVar database, have been identified in patients with syndromes associated with decreased Nav1.5 activity and are classified as VUSs (Table 2). An exception is genetic variant R219Q, which is listed in the ClinVar database as likely benign (Table 2). We propose that the long side chain of glutamine R219Q would form an H-bond with E161 in the resting VSD_I_. And although an H-bond is usually weaker than a salt bridge, the contact would still provide a stabilizing contribution to the resting-state VSD_I_.

We also found in the ClinVar database eleven mutations of R1_S4_I_ or E1_S2_I_ in five paralogs of the Nav1.5 channel (Nav1.1, Nav1.6, Nav1.7, Nav1.8, and Nav1.9). Two of these variants are reported as pathogenic, and nine variants as VUSs (Table 3). We suggest that salt bridge elimination in these variants is a possible mechanism of the channel dysfunction. Functional studies are necessary to estimate the likely pathogenicity of the VUSs. Our data suggest that such functional studies are promising and would allow the VUSs to be reclassified as pathogenic/likely pathogenic variants. Such a reclassification would be important for diagnostics of arrhythmias and the choice of treatment.

## 4. Materials and Methods

### 4.1. Structural Analyses and Modeling

The AlphaFold 3 (AF3) server [31] (https://golgi.sandbox.google.com/ was used to generate models of VSD_I_ in the WT channel Nav1.5 and mutant channels R219E/C/H/P. Structures were visualized and 3D aligned using PyMol (Schrödinger, New York, NY, USA, v.099). Molecular modeling was performed with the ZMM program (www.zmmsoft.ca) as described, e.g., in [32]. Briefly, energy calculations were performed using the AMBER force field [33] with environment and distance-dependent dielectric function [34]. The energetically optimal structures were predicted with the method of Monte Carlo (MC) energy minimization [35]. MC trajectories were terminated when the last 100th energy minimization did not improve the channel energy.

In silico deactivation of VSD_I_ was performed as described in [36,37]. Four CA atoms of voltage-sensing basic residues in helix S4_I_ (R1, R2, R3, and K4) were progressively downshifted with steps of 0.5 Å. At each step, the energy was MC-minimized with the CA atoms permitted to move freely within the plane normal to the pore axis, but not to leave the plane. To prevent refolding of VSD_I_, at each step of the downshifting trajectory, CA atoms in helices S1, S2, and S3 were allowed to deviate penalty-free from the starting position up to 4 Å, but at larger deviations, the energy penalty of 10 kcal mol^−1^ Å^−2^ was imposed.

CryoEM structures and AF3 models of sodium channels were 3D aligned by minimizing the root mean square deviations of C^α^ atoms in P1 helices from matching atoms in the Kv1.2-Kv2.1 channel (PDB code: 2R9R), the first eukaryotic P-loop potassium channels whose crystal structure was obtained with the resolution below 2.5 Å [38]. Such alignment shows not only deformations within VSDs, but also shifts of VSDs over the pore domain.

### 4.2. Site-Directed Mutagenesis and Heterologous Expression System Analyses and Modeling

Vector pcDNA3.1 with the WT hNav1.5 and GFP (hH1-pcDNA3.1) was kindly provided by Prof. Hugues Abriel (Institute of Biochemistry and Molecular Medicine, University of Bern, Bern, Switzerland). Site-directed mutagenesis was carried out by PCR amplification with overlapping primers complementary to the gene region containing the nucleotide substitution leading to R219E. Then, we eliminated the template WT cDNA via DpnI (New England Biolabs, Ipswich, MA, USA) restriction (overnight) and performed the chemical bacterial transformation using XL1-blue strain (Eurogene, Moscow, Russia). Next, we extracted mutated cDNA and verified mutagenesis via Sanger sequencing.

hH1-pcDNA3.1 (1 μg) or R219E-pcDNA3.1 (1 μg) were transfected into HEK293-T cells growing on 3 cm dishes using 1 mg/mL aqueous solution of linear polyethylenimine hydrochloride (PEI, molecular weight 40,000, Polysciences, Warrington, PA, USA) at a 2:1 v/m ratio with pDNA. On the first day, cells were seeded in 3 cm dishes, and on the second day, transfection was performed. The transfection mix (1 μg hH1-pcDNA3.1 (WT or R219E), 0.1 μg GFP, 2.2 μL PEI, and 1 mL DMEM medium without supplements) was incubated for 15 min at room temperature and then added to the cell culture. GFP was used as a marker of transfection efficiency and analyzed using fluorescent microscopy (Supplemental Figure S1). Cells were maintained in DMEM supplemented with 2 mM glutamine, 100 U/mL penicillin, and 100 μg/mL streptomycin (Thermo Fisher Scientific, Waltham, MA, USA) in a CO_2_ incubator at +37 °C for 24 h, and then seeded onto glass slides coated with poly-lysine (Sigma Aldrich, Burlington, MA, USA) for subsequent electrophysiological experiments.

### 4.3. Electrophysiology

The sodium current (I_Na_) was recorded using the patch-clamp method in the whole-cell configuration. All measurements were carried out at room temperature. The extracellular solution contained (mmol/L): 140 NaCl, 1 MgCl_2_, 1.8 CaCl_2_, 10 HEPES, and 10 glucose (pH 7.4 CsOH). The intracellular solution contained (mmol/L): 130 CsCl, 10 NaCl, 10 EGTA, and 10 HEPES (pH 7.3 CsOH). Glass microelectrodes were made from borosilicate glass using a P-1000 puller (Sutter Instrument, Novato, CA, USA). The electrode resistance varied from 2 to 3.5 MΩ. Series resistance was compensated by 75–80%. Data acquisition was carried out using an Axopatch 200B amplifier and Clampfit 10.3 software (Molecular Devices, San Jose, CA, USA). Currents were recorded at a frequency of 20 kHz, and low-pass filtering was carried out at a frequency of 5 kHz using an analog-to-digital interface (Digidata 1440A data acquisition system, Molecular Devices, San Jose, CA, USA). At least 3 independent transfections were used for electrophysiological recordings.

### 4.4. Data Analysis

The holding potential was −100 mV. The protocols used in the experiments are schematically represented in the figures. To record the current–voltage characteristics, we used the following protocol, with depolarizing pulses from −80 to 60 mV for 40 ms and a step of 5 mV at a frequency of 1 Hz. The current density at each test potential was estimated by normalizing INa to the cell capacitance.

To access the voltage dependency of the peak current advent, we analyzed current traces at potentials from −50 to +25 mV. The pulse start was considered as the zero time. We normalized each current trace to its maximal value and estimated time to the peak current. The latter was used as the initial point to analyze the inactivation decay. To simplify the analysis, we used 50% of the decay time instead of mono-exponential fitting. The decay time constant in the case of mono-exponential or bi-exponential fitting is highly variable and depends on the start and end points in the analyzed region, making difficult to standardize this parameter. We suggest using the time when the current returns to the level of 50% of maximum current (current decay 50%).

To access the steady-state activation, the maximal *I_Na_* at each voltage value was recorded, and the corresponding conductance was calculated using the following equation:

(1) $$\frac{G}{G_{max}}=\frac{I_{Na}}{V-V_{rev}}$$

where *G* is the conductance (Sm), *G_max_* is the maximum conductivity for Na^+^ ions, *I_Na_* is the sodium current (pA), *V* is the membrane potential (mV), and *V_rev_* is the reversal potential.

Next, we plotted the curve of steady-state activation of Nav1.5 (the voltage-dependence of normalized conductivity). The curves were approximated using the Boltzmann function:

(2) $$\frac{G}{G_{max}}=\frac{1}{1+\frac{\exp{(V}_{1/2}-V)}{k}}$$

where *G* is the conductance for Na^+^, *G_max_* is the maximum conductivity for Na^+^ ions, *V* is the membrane potential, *V*_1/2_ is the potential at which 50% of channels are activated, and *k* is the slope factor.

The voltage dependence of steady-state inactivation was measured using a two-step protocol with a 500 ms first pulse varying from −120 to 10 mV in 5 mV steps and a −15 mV test pulse lasting 20 ms. A curve of steady-state inactivation was plotted as the dependence of normalized *I_Na_* in response to the first testing voltage pulse and approximated using the Boltzmann function:

(3) $$\frac{I_{Na}}{I_{max}}=\frac{1}{1+\frac{\exp{(V}_{1/2}-V)}{k}}$$

where *I_Na_* is the sodium current, *I_max_* is the peak current, *V* is the membrane potential, *V*_1/2_ is the potential at which 50% of channels are inactivated, and *k* is the slope factor. The voltage dependence of fast inactivation was assessed in a similar way, but a protocol with a first-pulse duration of 20 ms was used. All results are presented as mean ± standard error (SEM). Statistical comparisons were performed using the unpaired Student test, with *p* < 0.05 considered statistically significant.

## 5. Conclusions

Glutamate substitution of the of the uppermost arginine R219 (R1) in helix S4 of the first voltage-sensing domain (VSD_I_) of the cardiac sodium channel hNav1.5 (R219E) caused negative shifts of the steady-state activation and inactivation and decelerated the recovery from inactivation. The enhanced activation of the mutant channel and its decelerated recovery from inactivation are consistent with the molecular modeling prediction on the salt bridge E161:R219 in the activated VSD_I_ and destabilization of the resting VSD_I_ due to electrostatic repulsion between glutamates E161 and R219E in the mutant channel. Elimination of the salt bridge is likely the atomic mechanism of dysfunction of ClinVar-reported disease variants of E161 or R219 for the hNav1.5 channel and mutations in analogous positions of sodium channel paralogs, suggesting that respective variants of unknown clinical significance are pathogenic/likely pathogenic variants.

## Figures and Tables

**Table 1:** Biophysical characteristics of Nav1.5 wildtype and R219E mutant channels ^a^.

Characteristics	Unit	WT	n ^b^	R219E	n ^b^	*p*
Peak current density	pA/pF	−426.5 ± 53.5	32	−299.3 ± 41.9	21	0.09
Steady-state activation	V_1/2_, mV K	−38.4 ± 1.2 5.4 ± 0.3	32	−46.1 ± 1.2 5.8 ± 0.3	21	<0.001 0.4
Steady-state inactivation	V_1/2_, mV K	−86.6 ± 1.4 −5.6 ± 0.2	24	−92.0 ± 1.2 5.5 ± 0.2	16	0.048 0.8
Steady-state fast inactivation	V_1/2_, mV K	−65.9 ± 2.0 8.5 ± 0.6	14	−77.0 ± 2.6 9.8 ± 0.3	14	0.0025 0.07
Recovery from inactivation	τ_fast,_ ms τ_slow_, ms	24.51 ± 3.2 241.1 ± 23.1	11	59.12 ± 13.4 404.7 ± 61.8	6	0.005 0.009

^a^ The data are presented as mean ± standard error (SEM). ^b^ Number of cells in the group.

**Table 2:** Nav1.5 genetic missense variants of E161 and R219 reported to the ClinVar database.

Variant	Clinical Significance	Phenotype
E161K NM_000335.5(SCN5A):c.481G>C	Conflicting interpretations of pathogenicity	BrS1 Progressive cardiac conduction disease Sick sinus syndrome
E161Q NM_000335.5(SCN5A):c.481G>A	Not provided	BrS1
R219C NM_000335.5(SCN5A):c.655C>T	Variant of unknown significance	BrS1
R219H NM_000335.5(SCN5A):c.656G>A	Conflicting interpretations of pathogenicity	BrS1 Dilated cardiomyopathy Sick sinus syndrome
R219P NM_000335.5(SCN5A):c.656G>C	Variant of unknown significance	Atrial fibrillation BrS1 Dilated cardiomyopathy LQT3 Progressive cardiac conduction disease Sick sinus syndrome
R219Q NM_001099404.2(SCN5A): c.656G>A	Likely benign	Arrhythmia

**Table 3:** ClinVar-reported missense variants of R1_S4_I_ or E1_S2_I_ in paralogs of Nav1.5.

Channel	Variant	Clinical Significance	Phenotype
Nav1.1	E158Q	Conflicting classifications of pathogenicity	Early infantile epileptic encephalopathy with suppression bursts
Nav1.6	R220C NM_014191.4 (SCN8A):c.658C>T	Uncertain significance	Early infantile epileptic encephalopathy with suppression bursts
Nav1.7	R214L NM_001365536.1 (SCN9A):c.641G>T	Uncertain significance	Neuropathy, hereditary sensory and autonomic, type 2A| Generalized epilepsy with febrile seizures plus, type 7
	R214Q NM_001365536.1 (SCN9A):c.641G>A	Uncertain significance	Inborn genetic diseases; generalized epilepsy with febrile seizures plus, type 7; neuropathy, hereditary sensory and autonomic, type 2A; generalized epilepsy with febrile seizures plus, type 7
	E156K NM_001365536.1 (SCN9A):c.466G>A	Uncertain significance	Generalized epilepsy with febrile seizures plus, type 7; neuropathy, hereditary sensory and autonomic, type 2A
Nav1.8	R215Q NM_006514.4 (SCN10A):c.644G>A	Uncertain significance	Episodic pain syndrome, familial, 2; Brugada syndrome
	R215W NM_006514.4 (SCN10A):c.643C>T	Uncertain significance	Cardiovascular phenotype; not provided
Nav1.9	R222H NM_001349253.2 (SCN11A):c.665G>A	Pathogenic	Hereditary sensory and autonomic neuropathy type 7; familial episodic pain syndrome with predominantly lower limb involvement
	R222C NM_001349253.2 (SCN11A):c.664C>T	Uncertain significance	Hereditary sensory and autonomic neuropathy type 7; familial episodic pain syndrome with predominantly lower limb involvement
	R222S	Pathogenic	Familial episodic pain syndrome with predominantly lower limb involvement
	E163D NM_001349253.2 (SCN11A):c.489G>T	Uncertain significance	Hereditary sensory and autonomic neuropathy type 7; familial episodic pain syndrome with predominantly lower limb involvement

## Data Availability

The original contributions presented in this study are included in the article/Supplementary Materials. Further inquiries can be directed to the corresponding author.

## Supplementary Materials

The following supporting information can be downloaded at: https://www.mdpi.com/article/10.3390/ijms26020712/s1.

## Abbreviations

AP: action potential; BrS1, Brugada syndrome type 1; Cryo-EM, Cryo-electron microscopy; INa, sodium current; LQT3, long QT syndrome 3 type; MC, Monte Carlo; Nav, voltage-gated sodium channel; Nav1.5, cardiac voltage-gated sodium channel; PDB, Protein Data Bank; PCR, polymerase chain reaction.
