# Supplementary material for: Charge Reversal of the Uppermost Arginine in Sliding Helix S4-I Affects Gating of Cardiac Sodium Channel
Source: Int J Mol Sci. 2025 Jan 16;26(2):712. doi: 10.3390/ijms26020712 (PMC11766011; doi:10.3390/ijms26020712)
Supplement: Supplementary file 1 [file ijms-26-00712-s001.zip › ijms-3360432-supplementary.pdf]

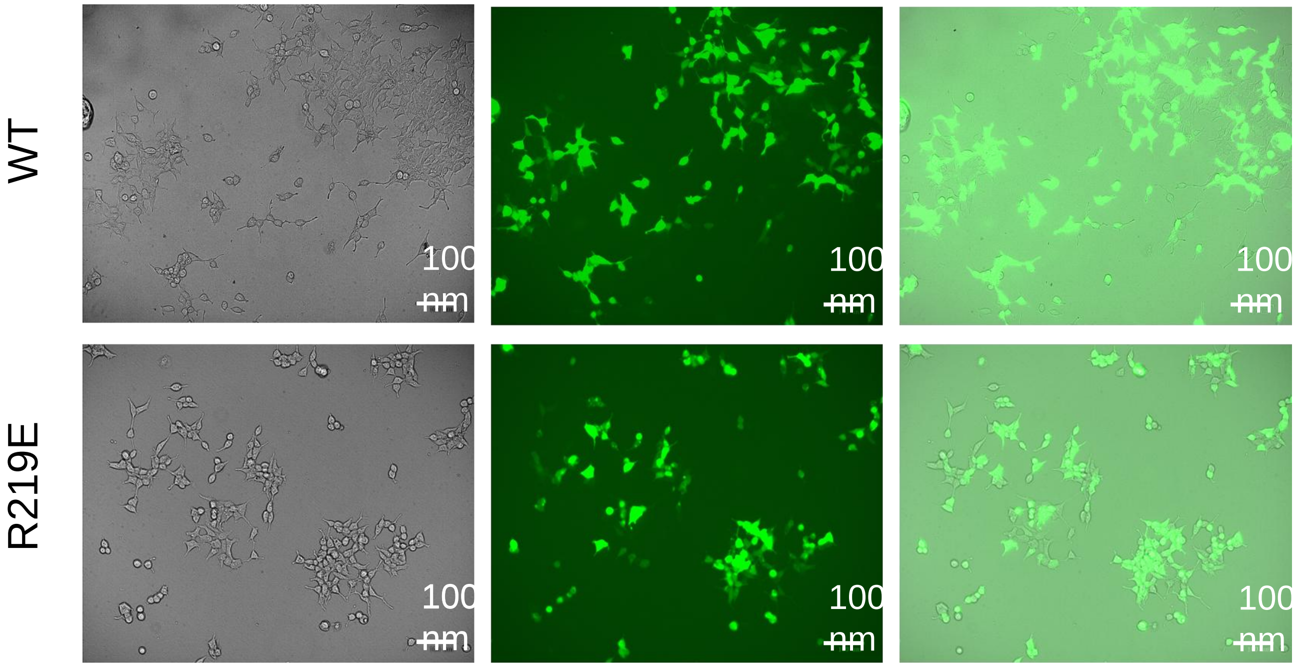

Figure S1. HEK293-T cells transfected with a genetic vector containing SCN5A-WT or SCN5A-R219E. GFP was used as a marker of transfection efficiency.
